# Artificial Intelligence‐Guided Identification of IGFBP7 as a Critical Indicator in Lactic Metabolism Determines Immunotherapy Response in Stomach Adenocarcinoma

**DOI:** 10.1111/jcmm.70301

**Published:** 2025-01-09

**Authors:** Minghua Wang, Xiaofei Guo, Xuyun Liu, Lei Huang, Chuang Yang

**Affiliations:** ^1^ Department of General Surgery The Second Affiliated Hospital of Harbin Medical University Harbin Heilongjiang China; ^2^ Department of Oncology The 962 Hospital of the Chinese People's Liberation Army Joint Logistic Support Force Harbin Heilongjiang China

**Keywords:** IGFBP7, immunotherapy, lactic metabolism, machine learning, STAD

## Abstract

Due to considerable tumour heterogeneity, stomach adenocarcinoma (STAD) has a poor prognosis and varies in response to treatment, making it one of the main causes of cancer‐related mortality globally. Recent data point to a significant role for metabolic reprogramming, namely dysregulated lactic acid metabolism, in the evolution of STAD and treatment resistance. This study used a series of artificial intelligence‐related approaches to identify IGFBP7, a Schlafen family member, as a critical factor in determining the response to immunotherapy and lactic acid metabolism in STAD patients. Computational analyses revealed that a high lactic metabolism (LM) state was associated with poor survival in STAD patients. Further biological network‐based investigations identified a key subnetwork closely linked to LM. Machine learning techniques, such as random forest and least absolute shrinkage and selection operator, highlighted IGFBP7 as a crucial indicator in STAD. Functional annotations showed that IGFBP7 expression was linked to important immune and inflammatory pathways. In vitro experiments demonstrated that silencing IGFBP7 suppressed cell proliferation and migration. Furthermore, heightened susceptibility to several chemotherapeutic drugs was linked to elevated IGFBP7 levels. In conclusion, this work sheds light on the mechanisms by which the lactate metabolism‐related indicator IGFBP7 affects the tumour immune milieu and the response to immunotherapy in STAD. The results point to IGFBP7 as a possible therapeutic target and predictive biomarker for the treatment of STAD.

## 1 Introduction

Gastric adenocarcinoma, or stomach adenocarcinoma (STAD), is a leading cause of cancer‐related mortality worldwide [1]. While recent advances in targeted and immune‐based therapies have improved outcomes for some STAD patients, the overall prognosis remains poor, with 5‐year survival rates below 30% [2]. A major challenge in STAD management is the significant inter‐ and intratumoral heterogeneity, contributing to variable treatment responses [3].

Emerging evidence suggests that metabolic reprogramming, a hallmark of cancer, plays a critical role in STAD progression and therapeutic resistance [4]. One key metabolic pathway implicated in STAD is lactic acid metabolism, where cancer cells exhibit increased glycolysis and lactate production even in the presence of oxygen (the Warburg effect) [5]. This lactic acidosis can profoundly impact the tumour microenvironment, suppressing antitumour immune responses and promoting immunotherapy resistance [6, 7]. Specifically, it has been noted that lactic acid enhances the expression of PD‐1 in regulatory T cells within tumour microenvironments that are highly glycolytic [8].

In this study, we employed a series of artificial intelligence‐related approaches to identify key regulatory factors within this metabolic programme to understand better the relationship between lactic metabolism and STAD immunotherapy response. Our analysis revealed IGFBP7, a little‐studied Schlafen family member, as a critical determinant of lactic acid metabolism and immunotherapy efficacy in STAD. Here, we report the mechanistic insights into how IGFBP7 modulates the tumour immune microenvironment and present its potential as a predictive biomarker and therapeutic target for STAD immunotherapy.

## 2 Materials and Methods

### 2.1 Data Collection and Procession

The TCGA (The Cancer Genome Atlas) STAD data set and GSE62254 [9, 10] (GEO, Gene Expression Omnibus) data set were used for all computational analysis. Following the RMA (Robust Multiarray Average) normalisation process [11], GSE62254 data set is comparable with TCGA STAD data set. TCGA STAD data set was used as the discovery set, while the GSE62254 data set was used as the validation set.

### 2.2 Computational Analysis

The lactic metabolism (LM) gene list was downloaded from the Molecular Signatures Database (MSigDB). LM score was calculated based on the LM gene list using Single Sample Gene Set Enrichment Analysis (ssGSEA). Weighted Correlation Network Analysis (WGCNA) was performed to determine the LM‐related subnetwork [12]. Soft threshold settings were implemented to guarantee a network topology without scaling and producing a TOM matrix. The parameter was set to a power of *β* = 10. Genes for the turquoise module were taken out for further exploration. Differentially expressed genes (DEGs) from the turquoise module genes between STAD and normal samples were identified using the R package limma, applying a cut‐off of abs(log2FC) > 1 and *p* value < 0.05 [13]. Univariate Cox regression analysis, along with machine learning methods Random Survival Forest (RSF) [14] and Least Absolute Shrinkage and Selection Operator (LASSO) [15], were used for the dimension reduction in DEGs. Univariate and multivariate Cox regression analysis was performed to determine the prognostic value of IGFBP7. The R package oncoPredict predicted drug responses related to IGFBP7 [16]. GISTIC 2.0 analysis was performed [17]. The R packages maftools generated the mutation landscape related to IGFBP7 [18, 19]. The immune cells by Tumour IMmune Estimation Resource (TIMER) and ssGSEA were calculated independently [20, 21, 22, 23].

### 2.3 In Vitro Validation on IGFBP7

The macrophage cell line THP‐1 and the STAD cell lines SU719 and SU601 were acquired from iCell. THP‐1 was polarised into M0 macrophage. IGFBP7 was silenced using three siRNA sequences (Forward CAATCCACTAACACTTTAGTT; Forward GCTGGTATCTCCTCTAAGTAA; and Forward GTCACTATGGAGTTCAAAGGA). STAD cells were treated with the most potent siRNA extract, total RNA. This RNA was then reverse‐transcribed into cDNA using a reverse transcriptase enzyme. The cDNA was subsequently used as a quantitative PCR (qPCR) amplification template. The abundance of target gene transcripts was determined using primers specific to each gene. The real‐time monitoring of the qPCR allowed for the precise quantification of mRNA levels. The relative expression was calculated using the 2^−ΔΔCt^ method, which involves normalisation to endogenous control genes.

The CCK‐8 assay measures cell viability and proliferation, where the absorbance values obtained are directly proportional to the number of viable cells. Higher absorbance indicates a greater number of viable cells.

The Transwell test was employed to evaluate STAD cells' capacity for migration. A Transwell plate with a permeable membrane was used to seed cells in the upper chamber. The number of cells moving through the membrane to the lower compartment was counted.

The Co‐culture Transwell assay was utilised to evaluate the ability of macrophages to migrate. In this experiment, STAD cells were positioned in the lower chamber of a Transwell plate, and macrophages were seeded in the top chamber. Next, the number of cells that moved from the top to the lower compartment across the membrane was measured.

### 2.4 Statistical Analysis

All statistical analyses were conducted using R. The comparison of normally distributed variables between the two groups was performed with the Student's *t*‐test, while the Wilcoxon test was used for non‐normally distributed data. A *p* value of less than 0.05 was considered statistically significant.

## 3 Results

### 3.1 WGCNA for LM‐Related Genes

**FIGURE 1:**
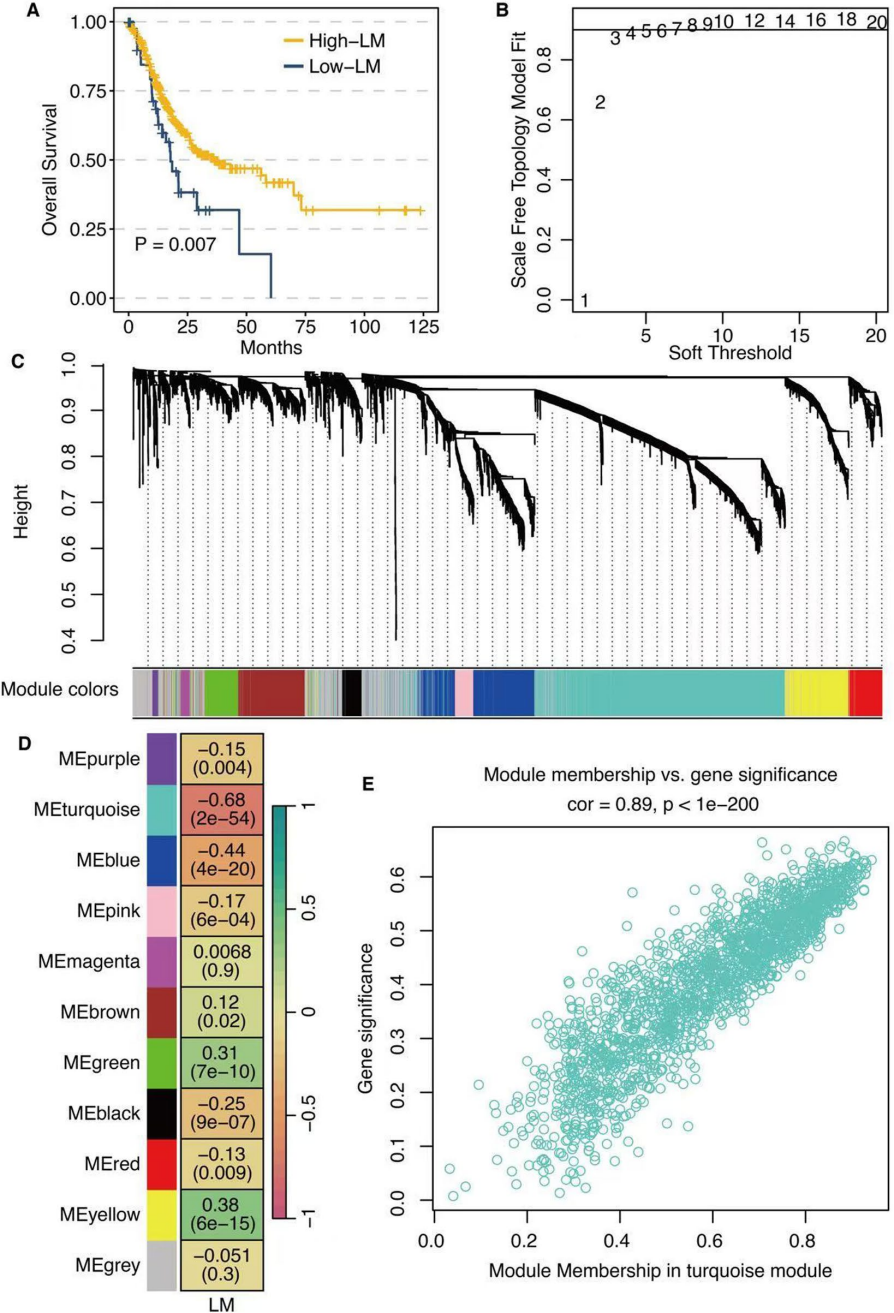
Weighted Correlation Network Analysis (WGCNA) for lactic metabolism (LM)‐related genes. (A) Survival curves of high (yellow) and low (blue) LM score groups in The Cancer Genome Atlas stomach adenocarcinoma data set. (B) Scale‐free topology model fit and soft threshold. (C) Module patterns of WGCNA. (D) Module–LM relationship. (E) Correlation between module membership and gene significance in the turquoise module.

**FIGURE 2:**
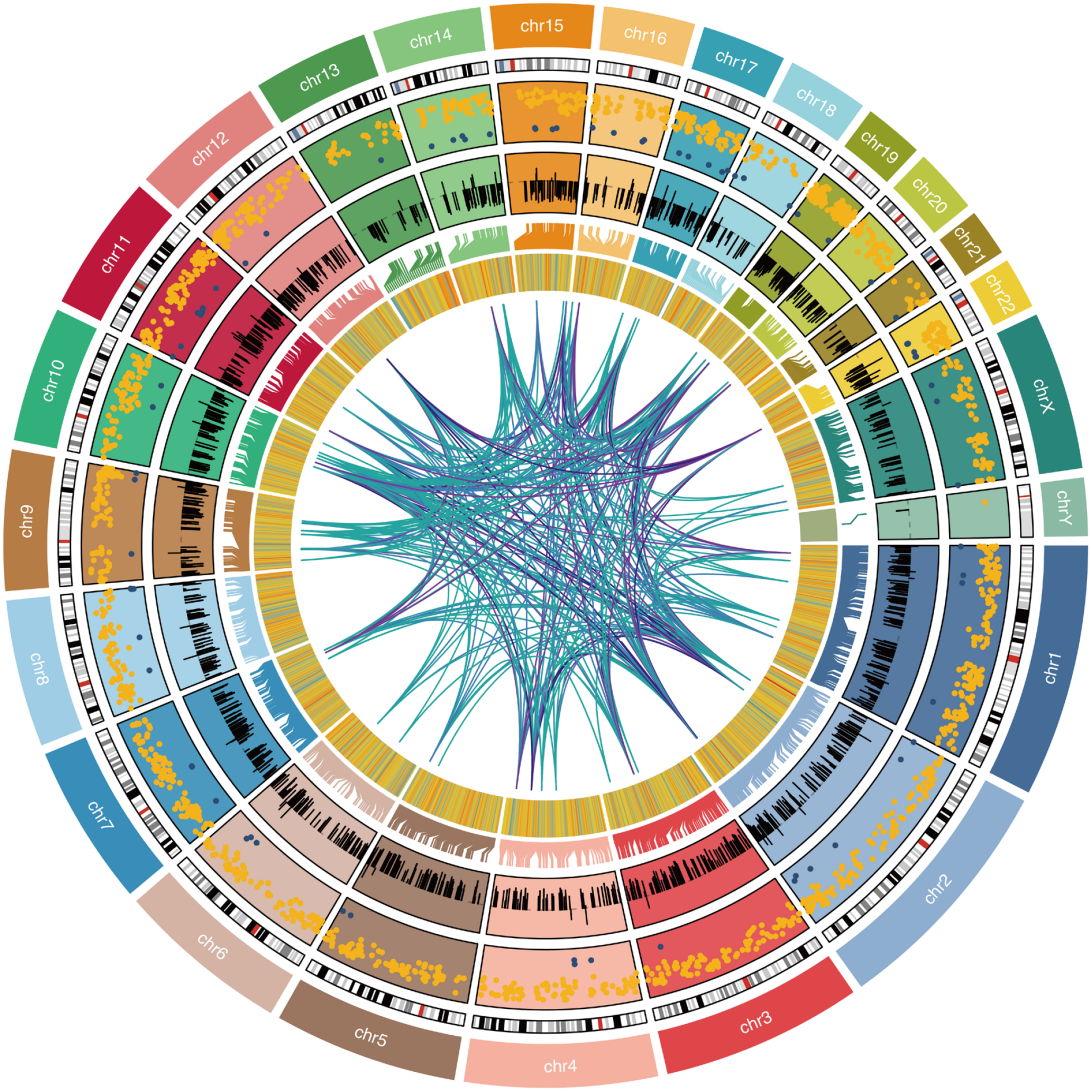
Chromosome distribution of module genes in the turquoise module.

The high LM score group was associated with significantly reduced survival time in the TCGA STAD data set (Figure 1A). Scale‐free topology model fit and soft threshold is shown in Figure 1B. Module patterns of WGCNA are shown in Figure 1C. Module–LM relationship revealed that the turquoise module was the most correlated module with LM (Figure 1D). A significant positive correlation was observed between module membership and gene significance in the turquoise module (Figure 1E). Chromosome distribution of module genes in the turquoise module is shown in Figure 2.

### 3.2 Machine Learning for Potent Genes

**FIGURE 3:**
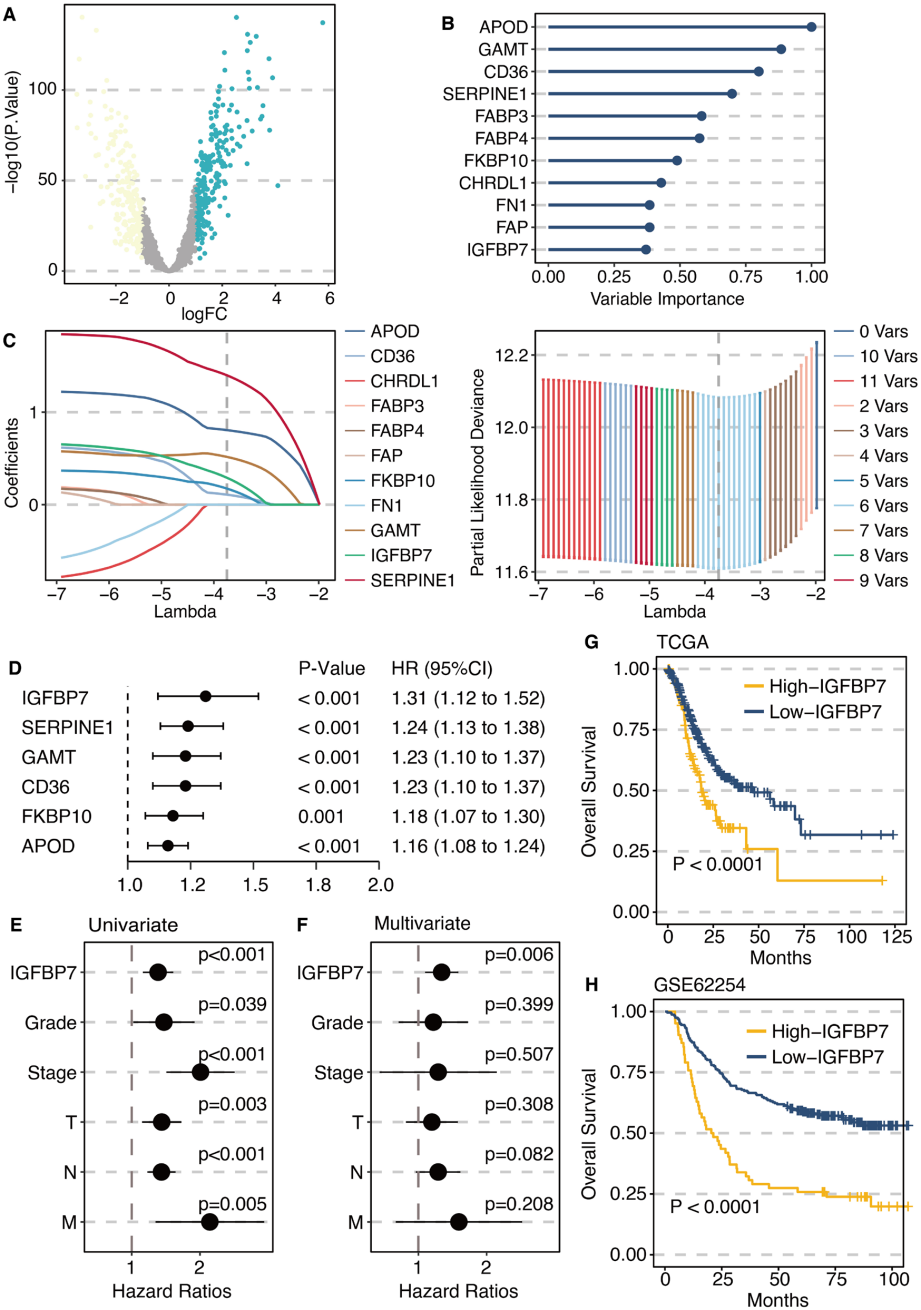
Machine learning for potent genes. (A) Differentially expressed genes (DEGs) from turquoise module genes between stomach adenocarcinoma and normal samples. (B) Random forest analysis on DEGs. (C) Least Absolute Shrinkage and Selection Operator regression analysis on DEGs. (D) Univariate Cox regression analysis on potent genes (IGFBP7, SERPINE1, GAMT, CD36, FKBP10 and APOD). (E) Univariate Cox regression analysis on IGFBP7 and clinical factors (Tumour grade, Tumour stage and TNM stage). (F) Multivariate Cox regression analysis on IGFBP7 and clinical factors (Tumour grade, Tumour stage, and TNM stage). (G) Survival curves of high (yellow) and low (blue) IGFBP7 groups in TCGA STAD data set. (H) Survival curves of high (yellow) and low (blue) IGFBP7 groups in GSE62254 dataset.

DEGs from turquoise module genes between STAD and normal samples are shown in Figure 3A. Random forest analysis was performed on DEGs to select the most potent genes (Figure 3B). LASSO regression analysis was also performed on DEGs to select the most potent genes (Figure 3C). Univariate Cox regression analysis on potent genes determined six prognostic genes including IGFBP7 (Figure 3D). Univariate Cox regression analysis on IGFBP7 and clinical factors confirmed the independent prognostic role of IGFBP7 (Figure 3E). Multivariate Cox regression analysis on IGFBP7 and clinical factors confirmed the independent prognostic role of IGFBP7 (Figure 3F). The high IGFBP7 group was associated with significantly reduced survival time in the TCGA STAD data set (Figure 3G). The high IGFBP7 group was associated with significantly reduced survival time in the GSE62254 data set (Figure 3H).

### 3.3 Mutation Characteristics of IGFBP7

**FIGURE 4:**
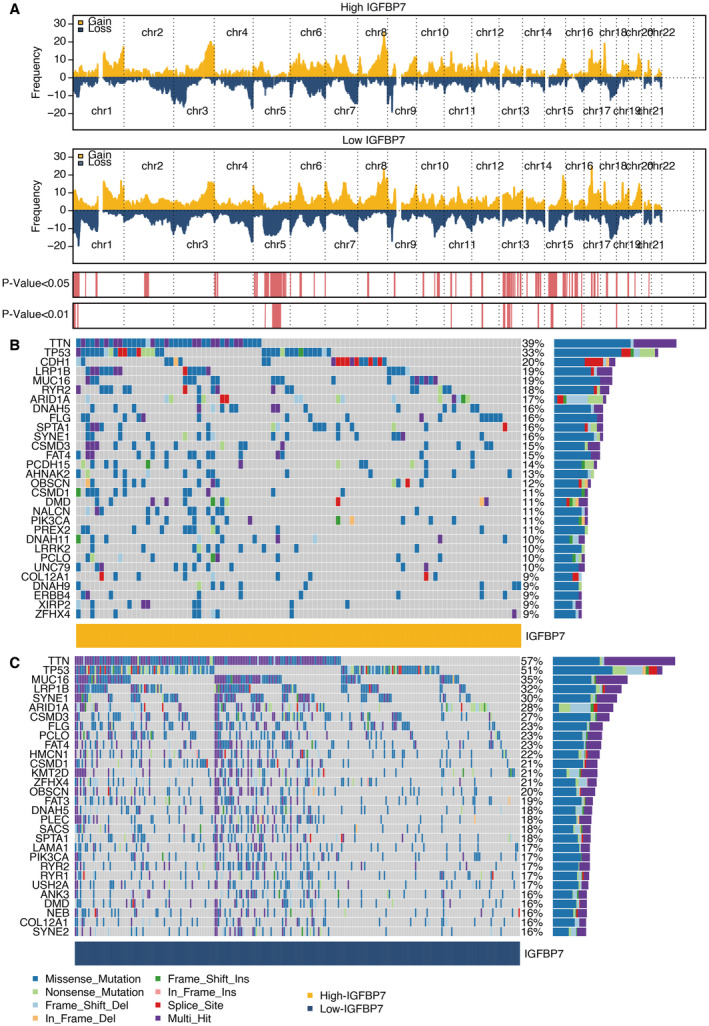
Mutation characteristics of IGFBP7. (A) CNV distribution in high (yellow) and low (blue) IGFBP7 groups in chromosomes. (B) Mutation landscape of the high IGFBP7 group. (C) Mutation landscape of the low IGFBP7 group.

CNV distribution in high and low IGFBP7 groups in chromosomes is shown in Figure 4A. Mutation landscape of the high IGFBP7 group showed that CDH1 was top ranker mutated gene (Figure 4B). The mutation landscape of the low IGFBP7 group showed that TP53 and TTN were top‐rank mutated genes (Figure 4C).

### 3.4 Functional Annotation and Drug Prediction of IGFBP7

**FIGURE 5:**
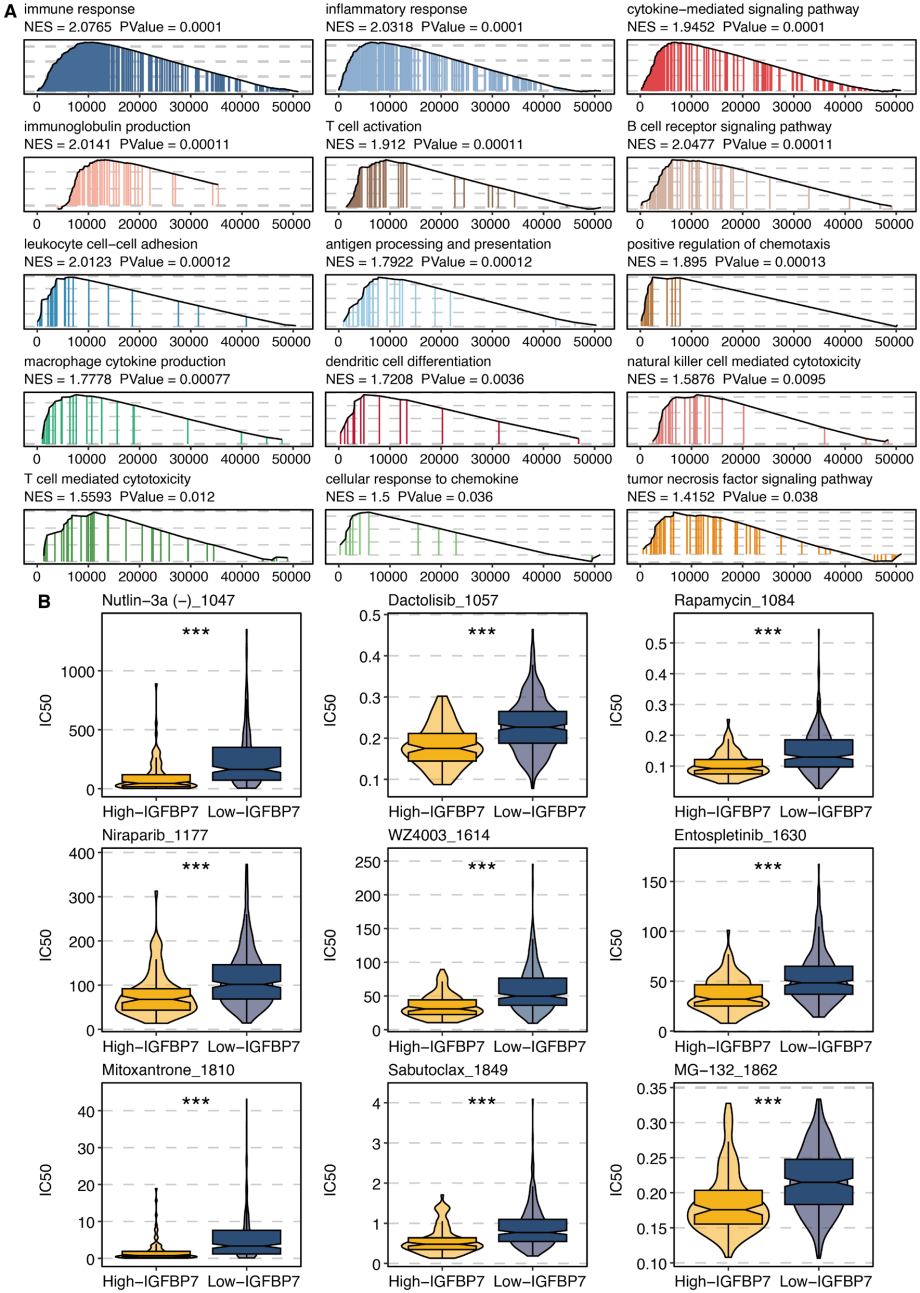
Functional annotation and drug prediction of IGFBP7. (A) Gene Set Enrichment Analysis on IGFBP7. (B) Estimated IC50 of chemotherapy drugs in high (yellow) and low (blue) IGFBP7 groups. ***p < 0.001.

GSEA on IGFBP7 showed that immune and inflammatory activity were significantly related to IGFBP7 (Figure 5A). Estimated IC50 of chemotherapy drugs, such as Nutlin‐3a, Dactolisib, Rapamycin, Niraparib, WZ4003, Entospletinib, Mitoxantrone, Sabutoclax and MG‐132, was significantly lower in high IGFBP7 group (Figure 5B).

### 3.5 In Vitro Validation on IGFBP7

**FIGURE 6:**
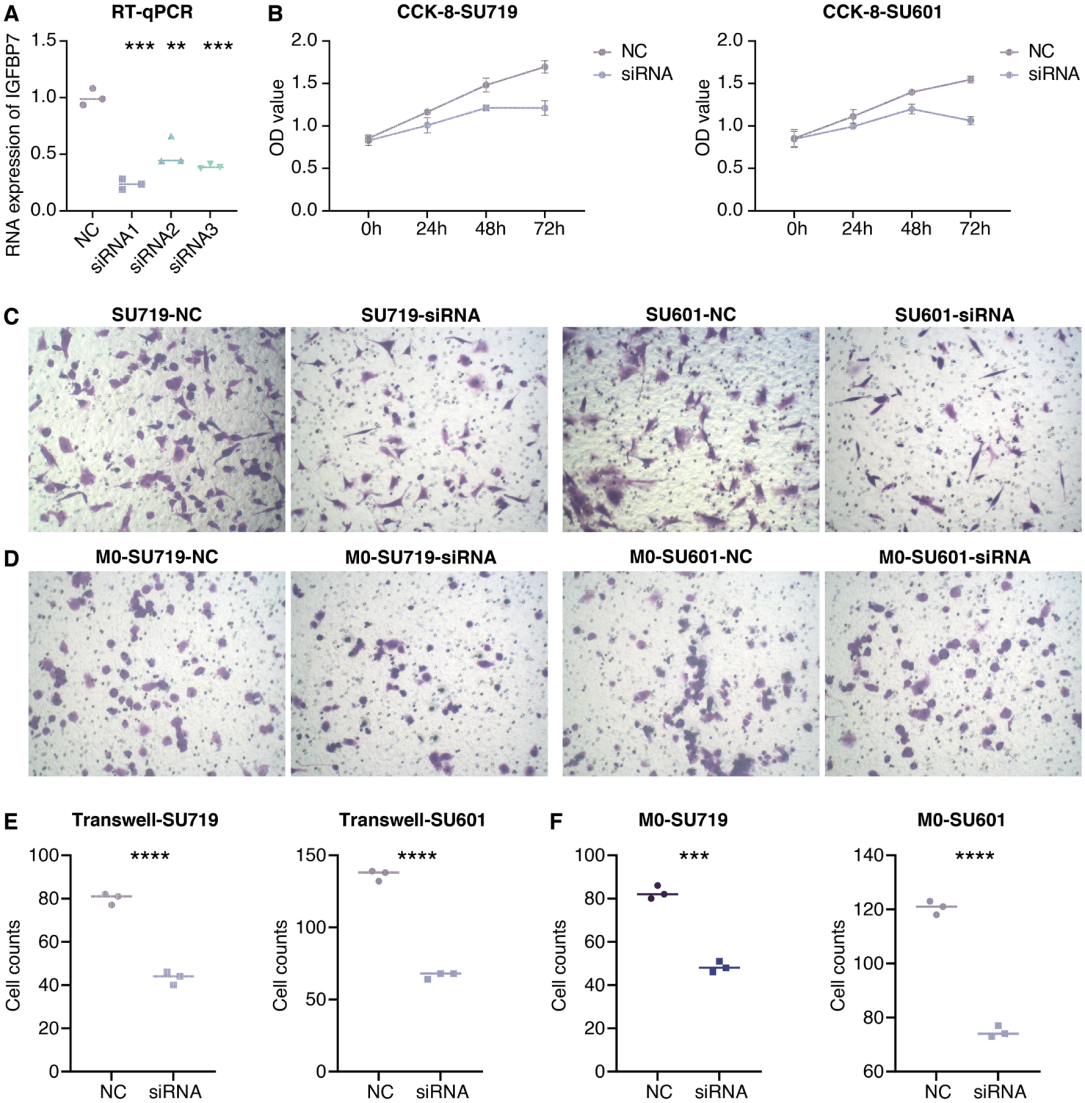
In vitro validation on IGFBP7. (A) RT‐qPCR assay shows the RNA expression of IGFBP7 in different groups in SU719 cells. (B) OD values of different groups in CCK‐8 assay in SU719 and SU601 cells. (C) Transwell assay shows the migrated STAD cells in different groups in SU719 and SU601 cells. (D) Co‐culture Transwell assay shows the migrated macrophages in different groups in SU719 and SU601 cells. (E) Statistical analysis of Transwell and Co‐culture Transwell assays in SU719 cells. (F) Statistical analysis of Transwell and Co‐culture Transwell assays in SU601 cells. **p < 0.01, ***p < 0.001, ****p < 0.0001.

RT‐qPCR assay showed that IGFBP7 expression was significantly suppressed in siRNA‐transfected groups in SU719 cells (Figure 6A). CCK‐8 assay showed that the proliferation ability of STAD cells was significantly suppressed in the siRNA‐transfected group in SU719 and SU601 cells (Figure 6B). Transwell assay showed that the migration ability of STAD cells was significantly suppressed in the siRNA‐transfected group in SU719 and SU601 cells (Figure 6C,E,F). Co‐culture Transwell assay showed that the migration ability of macrophages was significantly suppressed in the siRNA‐transfected group in SU719 and SU601 cells (Figure 6D–F).

### 3.6 Immunological Features of IGFBP7

**FIGURE 7:**
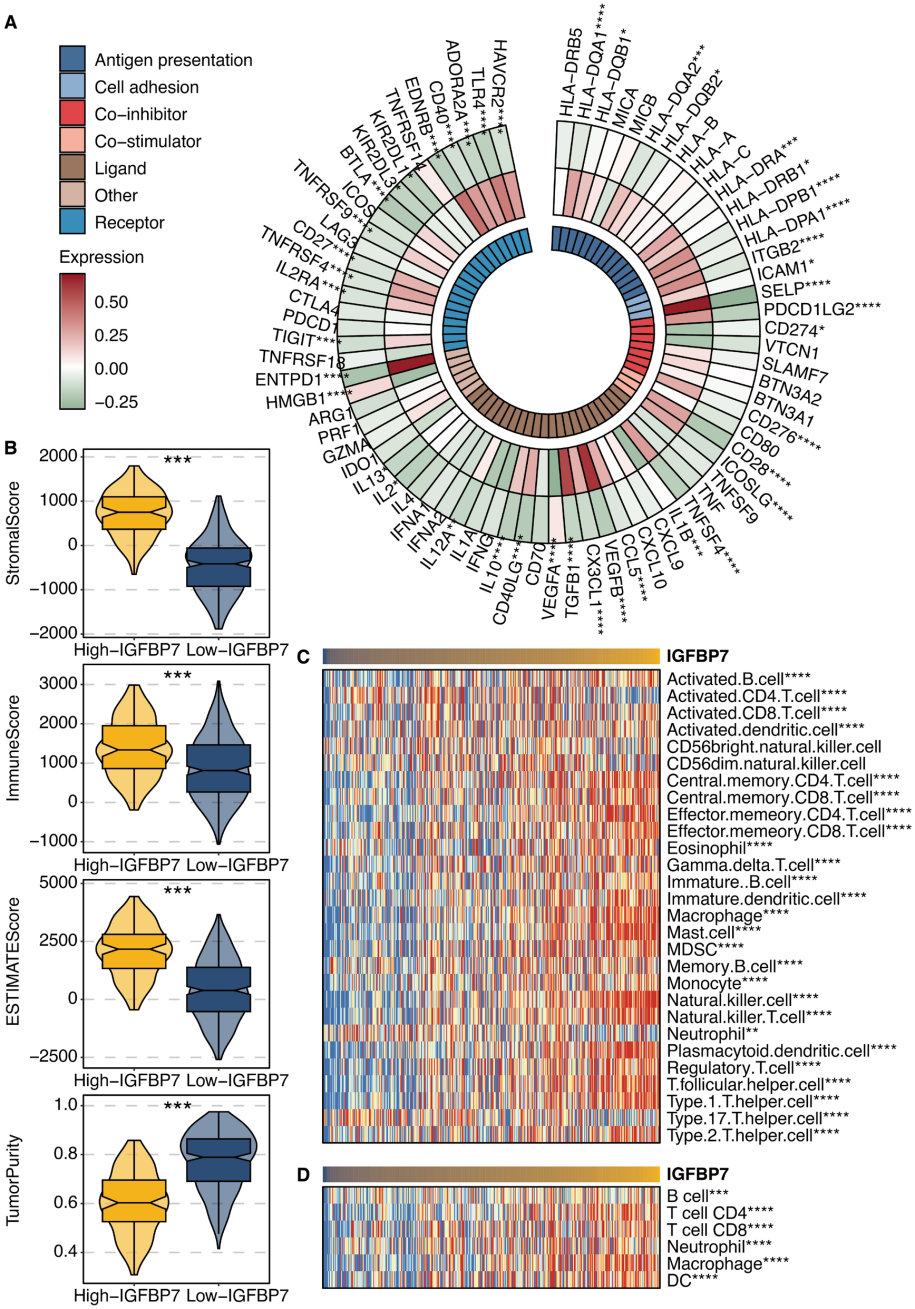
Immune features of IGFBP7. (A) Correlation between IGFBP7 and immune modulators. (B) Levels of microenvironment scores in high (yellow) and low (blue) IGFBP7 groups. (C) Correlation between IGFBP7 and ssGSEA‐based immune cells. (D) Correlation between IGFBP7 and TIMER‐based immune cells. *p < 0.05, **p < 0.01, ***p < 0.001. ****p < 0.0001.

**FIGURE 8:**
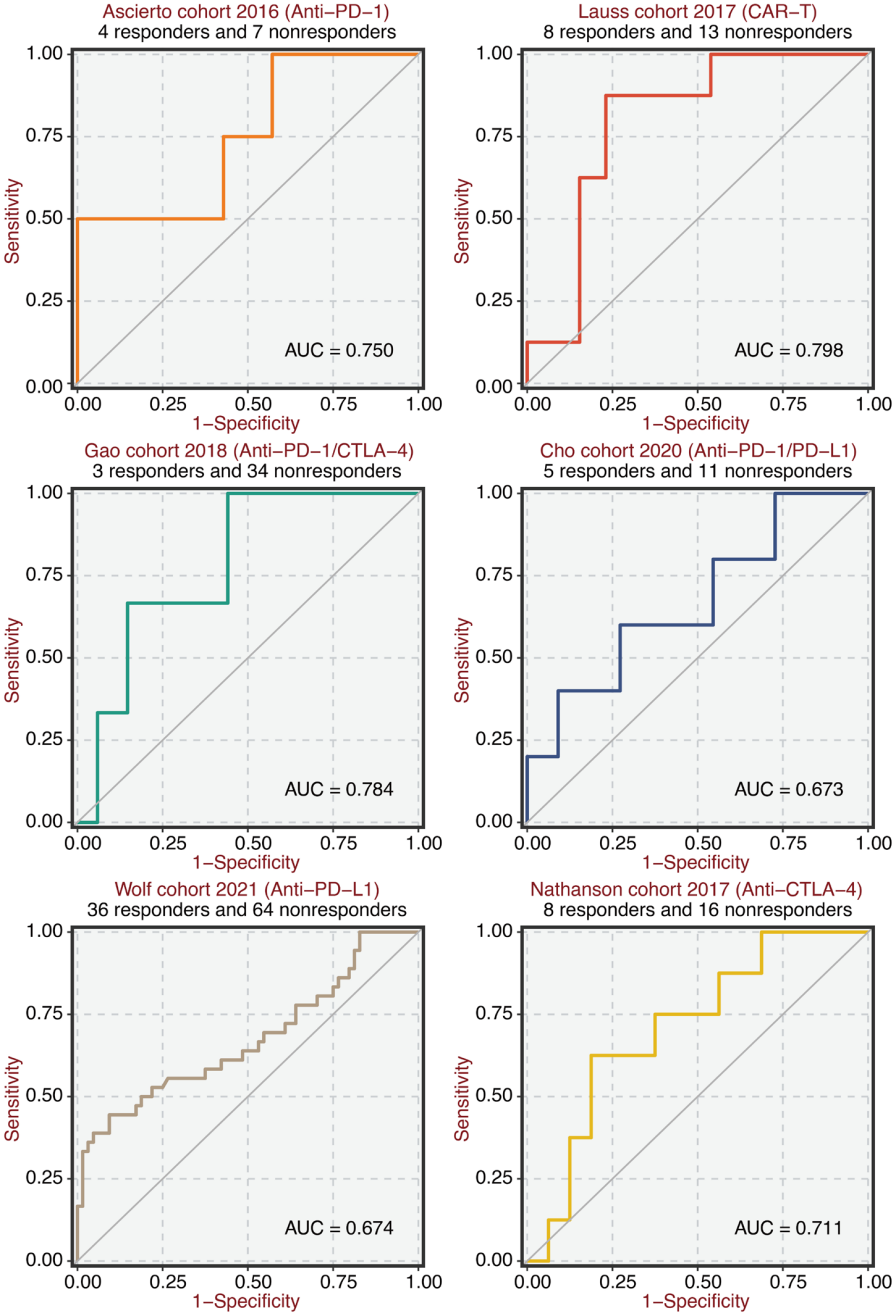
Immunotherapy prediction of IGFBP7. ROC plot shows the predictive value of IGFBP7 in six immunotherapy cohorts.

A significant positive correlation was observed between IGFBP7 and immune modulators in Figure 7A. Microenvironment scores were significantly higher in the high IGFBP7 group (Figure 7B). A significant positive correlation was observed between IGFBP7 and ssGSEA‐based immune cells (Figure 7C). A significant positive correlation was observed between IGFBP7 and TIMER‐based immune cells (Figure 7D). Next, the predictive value of IGFBP7 was confirmed in six immunotherapy cohorts, including anti‐PD‐1, anti‐PD‐L1, CAR‐T and anti‐CTLA‐4 (Figure 8).

## 4 Discussion

In this study, we employed a comprehensive machine learning approach to uncover the critical role of IGFBP7 in lactic acid metabolism and immunotherapy response in STAD. Our results demonstrate that high IGFBP7 expression is associated with poor prognosis and reduced survival in STAD patients. Importantly, we provide mechanistic insights into how IGFBP7 modulates the tumour immune microenvironment to mediate immunotherapy resistance.

Through WGCNA, we identified the turquoise module as the most significantly correlated with lactic acid metabolism in STAD. WGCNA is a powerful system biology tool that can uncover the intricate relationships between genes and their associated biological pathways [24]. Unlike traditional differential expression analysis, which examines genes individually, WGCNA considers the complex interconnectivity between genes and their collective influence on cellular processes. This holistic perspective allows for the identification of functionally relevant gene modules that are highly correlated with specific phenotypes, such as lactic acid metabolism in the case of STAD. Further machine learning analysis revealed IGFBP7 as a key regulator within this metabolic programme [25]. Random forest is an ensemble learning algorithm that constructs multiple decision trees and combines their outputs to make predictions [26]. This technique offers several advantages over traditional statistical models. First, random forest is highly effective in capturing complex, nonlinear relationships between predictor variables and the outcome of interest. This is particularly useful in cancer, where the underlying biology often involves intricate, multifactorial interactions. Second, random forest is inherently resistant to overfitting, as it generates an ensemble of models and aggregates their predictions, improving the overall robustness and generalisability of the results. LASSO regression is another powerful machine learning technique we employed in this study [27]. Machine learning has been widely applied in cancer research, especially cancer immunotherapy [25]. Combining the best aspects of WGCNA, random forest and LASSO, our study was able to systematically dissect the complex relationships between lactic metabolism, the immune microenvironment and immunotherapy response in STAD. LASSO is a type of penalised regression that can effectively perform feature selection by shrinking the coefficients of less important variables towards zero while retaining the most informative predictors. This method is especially advantageous when dealing with high‐dimensional data, as it can identify the smallest subset of genes that are most predictive of the outcome, greatly improving the interpretability and clinical applicability of the findings. This integrated, machine learning‐driven approach [28] allowed us to pinpoint IGFBP7 as a key regulator, highlighting its potential as a predictive biomarker and therapeutic target for improving STAD management. IGFBP7 is a secreted protein that has been extensively studied in the context of cancer biology [29]. While the exact mechanisms by which IGFBP7 influences tumour progression and treatment response are complex and context‐dependent, emerging evidence suggests that it plays a multifaceted role in cancer development, progression and immunotherapy [29, 30]. Functional annotation showed that IGFBP7 is closely associated with immune and inflammatory pathways, suggesting its importance in shaping the tumour immune landscape.

Our in vitro experiments confirmed the crucial role of IGFBP7 in regulating STAD cell proliferation and migration. Silencing IGFBP7 expression significantly impaired these malignant cellular functions, further validating its oncogenic role. More importantly, we demonstrated that IGFBP7 modulates the crosstalk between STAD cells and macrophages, a key component of the tumour microenvironment. IGFBP7 knockdown disrupted the migration of macrophages towards STAD cells, indicating its ability to suppress antitumour immune responses.

The mutation landscape analysis revealed distinct mutational patterns between high and low IGFBP7 expression groups. Notably, the high IGFBP7 group was characterised by a high frequency of CDH1 mutations, associated with increased lactic acid production and immune evasion in STAD [31, 32]. In contrast, the low IGFBP7 group exhibited a high prevalence of TP53 and TTN mutations, commonly observed in STAD [33, 34]. These findings suggest that IGFBP7 may interact with specific genetic alterations to shape the tumour metabolic and immune microenvironment.

Furthermore, our drug response prediction analysis identified several chemotherapeutic agents, including Nutlin‐3a, Dactolisib and Rapamycin, potentially more effective in STAD tumours with high IGFBP7 expression. This highlights the potential of IGFBP7 as a predictive biomarker for tailoring treatment strategies in STAD [35].

## 5 Conclusion

Our thorough investigation concludes by demonstrating the crucial function of IGFBP7 in controlling the immunological milieu surrounding tumours and lactic acid metabolism, eventually leading to immunotherapy resistance in STAD. These results imply that IGFBP7 might be a useful therapeutic target and predictive biomarker to enhance the clinical care of STAD patients. There are also limitations in the study. The study does not include clinical trial data to validate the predictive value of IGFBP7 in real‐world treatment settings, limiting the applicability of findings. While in vitro experiments support the findings, they may not fully replicate the complex in vivo tumour microenvironment. Results from cell lines may not reflect the behaviour of primary tumours. More research is necessary to confirm the mechanistic insights and investigate the therapeutic uses of IGFBP7 targeting STAD.

## Author Contributions

**Minghua Wang:** conceptualization (equal), data curation (equal). **Xiaofei Guo:** formal analysis (equal), software (equal). **Xuyun Liu:** methodology (equal). **Lei Huang:** methodology (equal), writing – original draft (equal). **Chuang Yang:** conceptualization (equal), methodology (equal), writing – original draft (equal).

## Consent

The authors have nothing to report.

## Conflicts of Interest

The authors declare no conflicts of interest.

## Data Availability

Data used in this work can be acquired from the TCGA and GEO databases.
